# Differences Between Amblyopic and Fellow Eyes in Optical Coherence Tomography: A Cohort from Jordanian Population

**DOI:** 10.3390/medicina61010072

**Published:** 2025-01-04

**Authors:** Noor M. Alqudah, Hasan Mohidat, Abdelwahab Aleshawi, Wedad Al-Dolat, Ali Alshami

**Affiliations:** 1 Division of Ophthalmology, Department of Special Surgery, Faculty of Medicine, Jordan University of Science and Technology, Irbid 22110, Jordan; abdelwahhabjamal@yahoo.com (A.A.); alshamiali195@gmail.com (A.A.); 2 Department of Ophthalmology, Museum District Eye Center, Houston, TX 77006, USA; h.mohidat@charlesgarciamd.com; 3 Department of Ophthalmology, Faculty of Medicine, Yarmouk University, Irbid 21163, Jordan; wedad.dolat@yu.edu.jo

**Keywords:** amblyopia, retinal nerve fiber layer, optical coherence tomography, macular thickness, visual impairment

## Abstract

*Background and Objectives*: Amblyopia is a condition where children undergo unilateral or bilateral vision loss due to a variety of disorders that impact the visual pathway. The assessment of retinal nerve fiber layer (RNFL) thickness in amblyopia has made optical coherence tomography (OCT) a useful technique for studying the pathophysiology of this condition. This study was conducted to assess OCT results for various forms of amblyopia, including macular thickness and peripapillary RNFL thickness. It is the first of its kind in Jordan. *Materials and Methods*: Sixty-one individuals, aged 14 to 67, who had been diagnosed with unilateral amblyopia participated in a prospective study. Both eyes underwent spectral-domain OCT imaging, and clinical and demographic information was gathered. Relationships between different types of amblyopia and OCT measurements were investigated. *Results*: Compared to the contralateral fellow eyes, the amblyopic eyes showed thicker fovea, and there were differences in foveal and macular thickness observed between the sexes. Individuals with anisometropic amblyopia exhibited a greater central macular thickness. While nasal optic nerve thickness was adversely correlated with age, inferior optic nerve thickness was considerably lower in amblyopia. *Conclusions*: This research indicates unique OCT traits in amblyopic eyes, which may have a role in amblyopia diagnosis and treatment. The prevention of long-term visual impairment depends heavily on early detection and care. More studies using larger cohorts and longitudinal designs are necessary to improve our knowledge of the pathogenesis of amblyopia and to provide the best possible clinical management approaches.

## 1. Introduction

In children, amblyopia is the most prevalent cause of unilateral or bilateral vision loss [1]. The primary mechanisms behind this are visual deprivation, strabismus, and anisometropia [1]. Amblyopia is characterized by a persistent and avoidable decrease in best-corrected visual acuity in the absence of any discernible disease of the eye or visual pathways [2]. However, it cannot be distinguished from other types of vision loss just based on visual characteristics. Rarely, subtle afferent pupillary abnormalities might arise in cases of severe amblyopia. Amblyopia can occasionally occur with visual loss that is directly brought on by an irreversible structural anomaly of the eye, such as coloboma or optic nerve hypoplasia [3]. Amblyopia affects 2–4% of children in North America and is the most prevalent cause of unilateral visual loss in children [4]. Additionally, it is the most common reason for unilateral visual impairment in people under the age of 60 years [5]. Possible factors contributing to the development of amblyopia include delayed development, premature birth, or a family history of amblyopia [6]. Uncorrected refractive errors appear to be a major contributing factor to amblyopia in certain populations. In Singapore, for instance, approximately 85% of amblyopia cases are linked to uncorrected refractive errors, while only 15% are associated with strabismus [1]. Visual deprivation, strabismus, or substantial anisometropia might cause atypical susceptible cortical development in the maturing visual pathway [6]. On the other hand, if the visual pathway is treated early during the developmental period, there is a higher likelihood of reversing amblyopia [7].

It has been concluded that amblyopia is a developmental condition that involves a pathophysiological shift from retinal ganglion cells to the visual brain [1]. Near the end of gestation [8], there is a sharp decrease in the cell density of the retinal ganglion cell layer during fetal development. The ganglion cell layer has a maximum population of 2.2–2.5 million cells between weeks 18 and 30 of gestation. The quantity thereafter drops to 1.5–1.7 million [8]. Furthermore, during pregnancy, the optic nerve’s axon count also falls. The estimated 3.7 million optic nerve axons reach their maximum at 16–17 weeks of gestation [8]. The retinal nerve fiber layer (RNFL) thickness in amblyopia may be impacted if the condition impedes the postnatal reduction in ganglion cells. Amblyopia arises during the phase of maturation and development of the neural network connecting the cerebral cortex and retina [8]. Therefore, the onset of amblyopia usually occurs in the first 2–3 years of life after birth, while it can also appear as late as 8–9 years after birth [9]. While the structures involved in amblyopia are still being investigated. Some research suggests that the visual deficit may originate in the lateral geniculate nucleus (LGN) showing dysfunction and atrophy of the LGN in individuals with amblyopia [10]. 

Red-free ophthalmoscopy, optical coherence tomography (OCT), and scanning laser polarimetry (SLP) are three methods that have been introduced to assess the thickness of the RNFL [11]. OCT was first introduced in 1991 by Huang and colleagues [12]. OCT is a non-invasive diagnostic method that captures a cross-sectional view of the retina. Interferometry is the concept utilized in OCT to create a cross-sectional map of the retina that is accurate to within at least 10–15 microns [13]. Due to the transparency of the eye, OCT has gained wide popularity as an ophthalmic diagnostic tool. Two types of OCT have been introduced: time-domain OCT and Fourier-domain OCT [14]. In time-domain OCT, the system acquires approximately 400 A-scans per second using six radial slices oriented 30 degrees apart. Because the slices are 30 degrees apart, care must be taken to avoid missing pathology between the slices [14]. Fourier-domain OCT has advantages over time-domain OCT regarding speed and the signal-to-noise ratio and has become the dominant type in the field of ophthalmology. Two types of Fourier-domain OCT exist: swept-source OCT and spectral-domain OCT, both of which require spectral interferograms, which are then Fourier-transformed to obtain an axial scan of the reflectance amplitude versus depth. Spectral-domain OCT produces approximately 20,000–40,000 A-scans per second. This increased scan rate and number diminish the likelihood of motion artifacts, enhance the resolution, and decrease the chance of missing lesions [13]. Swept-source OCT uses a wavelength-sweeping laser and dual-balanced photodetector, allowing for faster acquisition speeds of 100,000–400,000 A-scans per second [15].

Amblyopia has been studied using both spectral-domain and temporal-domain OCT. Spectral-domain OCT (SD-OCT) is the most often utilized subtype of OCT in clinical settings and amblyopia research [16]. OCT measures the RNFL thickness with non-contact and non-intrusive procedures. It is presumed that the RNFL thicknesses determined histologically and by OCT are equivalent [17]. OCT is based on near-infrared interferometry; therefore, light variations in nuclear sclerotic cataract density do not affect the thickness measurement, nor does it depend on the refractive status or axial length of the eye [17]. OCT has several applications, and it has been widely utilized in the anterior-segment imaging of the iridocorneal angle, with systems like time-domain, spectrum-domain, and swept-source OCT also being used to aid in diagnoses and enable detailed visualization of ocular outflow structures, including the trabecular meshwork, Schlemm’s canal, collector channels, and vessels in the corneoscleral limbus. OCT also enables ophthalmologists to directly observe anatomical features relevant to ocular fluid drainage and potential surgical sites in glaucoma cases [12,18].

To our knowledge, there have been no previous studies on OCT findings in amblyopia in Jordan. Therefore, in this study, we aim to analyze OCT results for several forms of amblyopia, including an analysis of the macular and peripapillary RNFL thicknesses and the anatomical, visual, and demographic characteristics of amblyopia.

## 2. Methods

### 2.1. Study Design

A prospective cross-sectional study was performed between November 2023 and April 2024 on patients aged between 14 and 67 who were diagnosed with unilateral amblyopia and invited to obtain an OCT scan of both eyes at King Abdullah University Hospital. Unilateral amblyopia is defined as a reduction in the best-corrected visual acuity of 2 or more lines of acuity between the eyes that is not attributed to a structural abnormality.

### 2.2. Data Collection

Data were collected for demographic variables including age and sex, as well as clinical variables, which included past medical and ocular history type and laterality of amblyopia. In addition, a comprehensive ophthalmic examination was performed for all patients, which included evaluations of uncorrected and best-corrected visual acuity, refraction, extraocular muscle motility and balance, strabismus, the anterior and posterior segments, and intraocular pressure. The OCT parameters analyzed included the central subfoveal thickness, the fovea minimum (the thinnest part in the foveal region), the whole-area volume, and the 4 peripapillary quadrants’ RNFL thicknesses. Sixty-one patients were enrolled in this study.

### 2.3. Inclusion Criteria

All cooperating patients (older than 12) with unilateral mild (visual acuity of 6/9 to 6/12), moderate (worse than 6/12 to 6/36), or severe (worse than 6/36) amblyopia were included. Strabismic, anisometropic, and meridional amblyopia with central fixation were included in this study, defined according to the American Academy of Ophthalmology. Unilateral amblyopia with distinct refractive errors between the eyes was classified as anisometropic amblyopia, typically associated with 1.0–1.5 D or more aniso-hyperopia, 2.0 D or more aniso-astigmatism, or 3.0–4.0 D or more aniso-myopia. Meridional amblyopia, caused by significant astigmatism (2.0–3.0 D or more), was identified when the patient presented with marked astigmatism [2]. Patients who had diabetic retinopathy or a history of macular diseases were excluded. Furthermore, reluctant patients, patients with poor or eccentric fixation, patients with an intellectual disability, and patients with any uncorrected structural abnormalities of the eye, were among those excluded according to the exclusion criteria. The contralateral fellow eyes of the participants were considered a control group. All patients had unilateral amblyopia with no observed abnormalities in the contralateral fellow eyes.

### 2.4. Ethical Considerations

The approval of the institutional review board (IRB) was obtained from Jordan University of Science and Technology (ID: 2023036). We certify that participant privacy was preserved and that confidentiality and anonymization were applied to the data. Each patient, as well as the parents or legal guardians of individuals under the age of 18, gave written informed permission. The 1964 Declaration of Helsinki, as well as its subsequent revision, established ethical guidelines that this study adhered to.

### 2.5. Examination Settings

The ocular examinations and the interpretation of the OCT results were performed by one consultant ophthalmologist and two skilled trainees. Snellen charts were used to quantify visual acuity, and the results were converted to the LogMAR system. The refractive status was assessed with an autorefractor (Nidek ARK-510A, Tokyo, Japan) both before and following cycloplegia using cyclopentolate 1%, and this step was followed by subjective refraction. Intraocular pressure (IOP) was assessed using Goldmann tonometry, and slit-lamp biomicroscopy (TopconSL-3D Slitlamp, Tokyo, Japan) was used to examine the anterior and posterior segments with the aid of a SuperField Volk lens.

Following the process of pupillary dilatation, all the eyes that met the criteria were analyzed using SD-OCT (Retinascan RS-3000; NIDEK, Gamagori, Japan). Both the macular and peripapillary regions were examined. Four radial scans, each measuring 6 mm in length, were positioned at the fovea in the macula at angles of 0°, 45°, 90°, and 135°. A circular scan with a diameter of 3.46 mm, centered on the optic disk, was performed in the peripapillary area using the “Disc Circle” option. The scans comprised 1024 A-scans, utilizing high-definition (50 HD) frame-enhancement software. This equipment is equipped with a light source that emits electromagnetic radiation with a wavelength of 880 nm. This instrument does not produce mirrored images like other SD-OCT instruments. The instrument has demonstrated excellent consistency and accuracy in measuring both healthy and diseased eyes. The Early Treatment Diabetic Retinopathy Study was used to acquire nine consecutive macular sectors located in the foveal center [19]. Retinal thickness was determined by measuring the distance between two interfaces at each location along the x-axis of the scan. The measurement of the central macular thickness was taken at a distance of 1 mm from the foveal center. A low-intensity light source was utilized for internal fixation, and the procedure was performed in a dimly lit environment. Images exhibiting a signal strength below 7/10, inadequate centering, motion artifacts, or dark areas were removed, ensuring a minimum of 2 images of acceptable quality.

### 2.6. Statistical Analysis

Continuous data were represented by means and their related standard deviations (SDs), whereas categorical variables were summarized using frequencies and percentages. To examine the relationship between demographic, clinical, and optic characteristics and amblyopia, a paired-sample *t*-test was utilized for continuous data. The McNemar test was employed to analyze paired samples for categorical variables. The association between OCT measurements and study factors was investigated using a linear regression model with a standardized mean estimate. Statistical significance was established when the *p*-value was less than 0.05. The statistical analyses were performed using R software (version 4.2.3, Vienna, Austria).

## 3. Results

### 3.1. Patient Demographic Characteristics

A total of 61 patients with amblyopia participated in the study, with a mean age of 36 years (standard deviation: 18), comprising 56% males and 44% females. The majority (70%) had no significant medical history, while 11% had hypertension, 6.6% had diabetes, and the remaining had asthma, myasthenia gravis, or hypothyroidism. An evaluation of the patients’ past ocular history revealed that 49% had no issues, 25% wore eyeglasses, and smaller percentages had undergone various ocular surgeries (including squint surgery, cataract surgery, and refractive surgery). Amblyopia affected the left eye in 56% of patients, with anisometropic amblyopia being the most common type (85%), followed by strabismic (13%) and meridional (1.7%) amblyopia. Table 1 shows the patients’ general characteristics.

### 3.2. Comparison with Contralateral Fellow Eyes

A comparison between the amblyopic and contralateral fellow eyes revealed significant differences. Amblyopia was more prevalent in the left eye compared to the fellow eyes (64% vs. 44%, *p* = 0.030). Additionally, the IOP was significantly lower in the amblyopic eyes (mean: 13.61 mmHg vs. 13.90 mmHg, *p* = 0.021), while the fovea thickness was significantly greater (mean: 220 μm vs. 209 μm, *p* = 0.047). The inferior optic nerve thickness was also significantly lower in the amblyopic eyes (mean: 127 μm vs. 134 μm, *p* = 0.026). The refractive errors seen in the amblyopia patients included hypermetropia reaching +6.5 and myopia reaching −12.0. Table 2 reveals the differences between the amblyopic and contralateral fellow eyes.

### 3.3. Linear Regression Analysis

The linear regression model revealed significant associations. The superior optic nerve thickness (SONT) exhibited a negative association with age (estimate: −0.48, *p* = 0.027) and a positive association with best-corrected visual acuity (BCVA) (estimate: 0.32, *p* = 0.054). The nasal optic nerve thickness (NONT) was negatively associated with age (estimate: −0.47, *p* = 0.023). Table 3 shows the linear regression analysis results for those variables.

### 3.4. Comparison of OCT Measures

**Figure 1:**
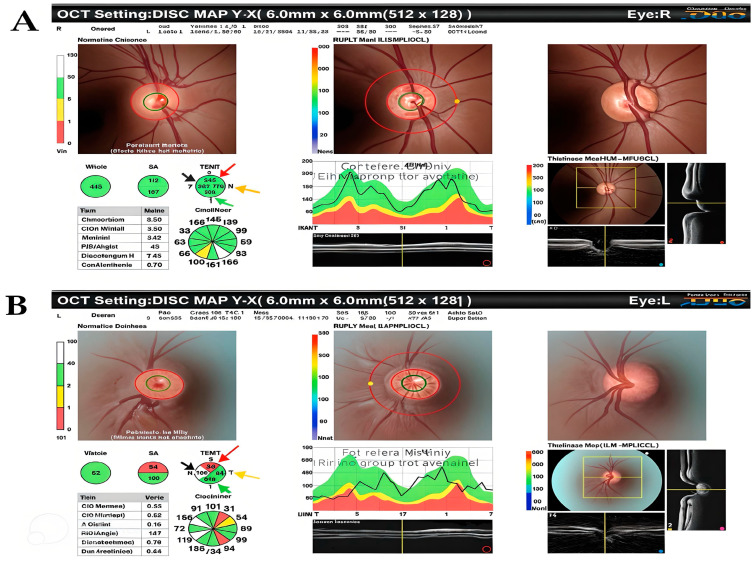
Optical coherence tomography of the optic nerve head and retinal nerve fiber layer in a 21-year-old male patient with amblyopia in the left eye shows the RNFL’s thickness in the inferior, superior, and nasal quadrants. The superior RNFL (red arrow), inferior RNFL (green arrow), nasal RNFL (black arrow), and temporal RNFL (yellow arrow) are shown. (**A**) Optical coherence tomography of the retinal nerve fiber layer in the right eye and (**B**) optical coherence tomography of the retinal nerve fiber layer in the left eye, showing a significantly noticeable decrease in the superior RNFL thickness in the left eye compared to the right eye.

Significant differences were observed in terms of OCT measures between study variables. Males showed greater central macular thickness (mean: 278.1 μm vs. 251.9 μm, *p* = 0.019), fovea thickness (mean: 228.6 μm vs. 207.3 μm, *p* = 0.007), whole-area volume (mean: 8.6 μm vs. 8.2 μm, *p* = 0.01), superior optic nerve thickness (mean: 131.8 μm vs. 112.1 μm, *p* = 0.004), and temporal optic nerve thickness (mean: 73.4 μm vs. 65.1 μm, *p* = 0.017) compared to females. Patients with left-sided amblyopia exhibited greater nasal optic nerve thickness (mean: 87.7 μm vs. 79.7 μm, *p* = 0.002). Furthermore, those with anisometropic amblyopia had greater central macular thickness (mean: 273.6 μm vs. 222.5 μm, *p* = 0.017). The severity of amblyopia (mild vs. moderate vs. severe) does not influence OCT parameters. In Table 4, the relationship between the OCT measures and the study variables is shown. Figure 1 shows the optic nerve head and retinal nerve fiber layer OCT with superior, inferior, nasal, and temporal quadrant thicknesses for a 21-year-old male patient with amblyopia in the left eye compared to the contralateral fellow eye.

## 4. Discussion

This is the first Jordanian study to examine retinal nerve layer thickness in amblyopia patients. It demonstrated that unilateral amblyopia leads to thicker fovea. Females had thinner foveal and macular thicknesses. Additionally, individuals with anisometropic amblyopia had a thicker central macular.

Studies have been conducted to evaluate the effectiveness of OCT in the identification, classification, and management of amblyopia [20,21]. The macular fovea of the myopic and hyperopic anisometropic amblyopic group was demonstrated by Kasem et al. to be thicker than that of the fellow group. Likewise, they found in their study that the foveal retina was thicker in the meridional amblyopic and strabismus amblyopic groups than in the contralateral eyes. Also, in deprivational amblyopia, the macular fovea was thicker than in the contralateral eye [20,21]. A study by Rajavi et al. demonstrated that anisometropic and strabismus amblyopic eyes had thicker macular foveal layers than contralateral amblyopic eyes. In addition, they proposed that the macular fovea and RNFL of the optic disk were thicker in hyperopic anisometropic amblyopia patients [22]. The same conclusion was reported in a meta-analysis by Li et al. showing that amblyopic eyes had a significantly greater foveal minimum thickness, mean foveal thickness, and mean macular thickness compared to the fellow eyes [23].

A comparative study by Andalib et al. also revealed that in anisometropic amblyopia, the macular thickness was significantly greater in the amblyopic eyes compared to the fellow eyes, while there was no significant difference in peripapillary nerve fiber layer thickness [24]. The lack of significant differences in papillary RNFL thickness in anisometropic amblyopia in their study could be attributed to the influence of refractive errors in RNFL measurements using OCT, as previous studies have shown a positive correlation between RNFL thickness and refractive error (with thicker RNFLs in more hyperopic eyes). Their study excluded patients with myopia or hyperopia greater than 5 diopters in either eye to minimize potential confounding effects [25,26]. These reports are in agreement with the results of the current study.

Hyperopia and axial length have been debated in relation to anisometropic amblyopia [27]. Anisometropic amblyopes may have hyperopic refractive errors and shorter axial lengths, signifying thicker retinal tissue, although other investigations have shown no significant changes. Arora et al. showed no significant difference in axial length between the amblyopic and fellow eyes in anisometropic amblyopia patients [27].

Numerous investigations, however, have discovered that there is no variation in thickness that would benefit amblyopic eyes. Walker et al. found no evidence of a significant difference in macular thickness or peripapillary RNFL thickness between the contralateral and amblyopic eyes [28,29]. Furthermore, Sloper et al. discovered no differences between children with normal eyes and those with amblyopic eyes in terms of the thicknesses of the RNFL and fovea [30].

Several histopathological studies have demonstrated that the amblyopic process can impact different levels of the visual pathway [3]. The amblyopic eye of a monkey with strabismic, anisometropic, or visual deprivation amblyopia showed a marked reduction in the number of cells in the lateral geniculate nucleus [30]. Anisometropic and strabismic amblyopia in humans share similarities in terms of their lateral geniculate nucleus results [28]. The role of the retina in amblyopia is a subject of debate [31]. Electroretinogram experiments in humans revealed a substantial reduction in response when pattern stimuli were shown to patients with amblyopia [32]. However, the foveal visual pigment density and the Stiles–Crawford effect did not indicate any impairment in the cone photoreceptors of amblyopic eyes [33]. Ikeda and Termain’s investigation revealed that in kittens with induced esotropia, there was a reduction in the spatial resolution of retinal ganglion cells, specifically in the centralis area of the deviated eye disease [34]. In the case of cats, the closure of the palpebral fissure or surgical strabismus did not lead to a decrease in ganglion cell resolution [35]. These hypotheses may contradict the findings of other publications that have consistently shown an increase in foveal thickness, including our own work. The variation in the outcomes of these investigations could be attributed to the differing experimental methodologies, ethnic backgrounds, or refractive anomalies investigated.

## 5. Conclusions

Numerous retinal and optic nerve discoveries were made throughout this examination of amblyopia. The foveal and central macular thicknesses were thinner in females. Patients with anisometropic amblyopia showed a thicker central macular. These results corroborate earlier studies on amblyopia that revealed changes to the retina and optic nerve. A significant portion of the population, particularly children, suffer from amblyopia. Early detection and treatment are necessary to prevent long-term visual impairment. OCT measurements of the retina and optic nerve may help in the diagnosis and treatment of amblyopia, mainly by excluding any underlying disease and classifying amblyopia according to our results. This study’s limited insights were caused by its small sample size and the absence of a non-amblyopic control group. Using larger cohorts and longitudinal techniques could help us better understand the pathophysiology and treatment of amblyopia. Notwithstanding these drawbacks, our results contribute to the literature on amblyopia by highlighting the necessity of thorough ocular examinations and targeted treatments in clinical practice.

## 6. Study Limitations

There are a few limitations to our study. The sample size is relatively small, but the number of participants in this study is comparable to other studies. Another limitation is the absence of a control group consisting of non-amblyopic individuals. However, we were able to utilize each patient’s contralateral fellow eye as a control. While the fellow eye was used for comparison in this study, it is important to note that previous research has indicated that the fellow eye in amblyopia may exhibit subtle structural changes and may not be entirely normal [36].

## Figures and Tables

**Table 1:** Demographic and clinical characteristics of included patients.

Characteristic	N = 61 ^1^
**Sex**	
Female	27 (44%)
Male	34 (56%)
Age (mean)	36 (18)
**Laterality**	
Left eye	34 (56%)
Right eye	27 (44%)
**Type of amblyopia**	
Anisometropic	52 (85%)
Meridional amblyopia	1 (1.7%)
Strabismic	8 (13%)

^1^ n (%); mean (SD).

**Table 2:** Comparison between amblyopic and contralateral fellow eyes and study variables.

Characteristic	Amblyopic Eye, N ^1^ = 61	Fellow Eye, N = 61	*p*-Value ^2^
**Laterality**			**0.030**
** Left eye**	39 (64%)	22 (36%)	
** Right eye**	22 (36%)	39 (64%)	
**UCVA**	0.59 (0.93)	0.75 (0.31)	0.238
**BCVA**	0.47 (0.50)	0.94 (0.15)	**<0.001**
**Lens status**			
** Clear**	47 (77%)	47 (77%)	
** Early cataract**	3 (4.9%)	5 (8.2%)	
** Mild cataract**	6 (9.8%)	6 (9.8%)	
** Pseudophakic**	5 (8.2%)	3 (4.9%)	
**Posterior segment exam**			-
** BRVO with macular edema**	0 (0%)	1 (1.6%)	
** Large myelinated nerve fiber layer over disk and macula**	1 (1.6%)	0 (0%)	
** Normal**	60 (98%)	60 (98%)	
**IOP**	13.61 (1.52)	13.90 (1.69)	**0.021**
**Central macular thickness**	267 (45)	266 (39)	0.832
**Fovea minimum**	220 (41)	209 (41)	0.047
**Whole-area volume**	8.42 (0.83)	8.56 (0.61)	0.192
**Superior optic nerve thickness**	123 (26)	127 (20)	0.338
**Inferior optic nerve thickness**	127 (26)	134 (20)	**0.026**
**Temporal optic nerve thickness**	69 (11)	72 (13)	0.139
**Nasal optic nerve thickness**	86 (18)	82 (14)	0.162

^1^ n (%); mean (SD) ^2^ McNemar test; paired student *T*-test. Abbreviations: UCVA, uncorrected visual acuity; BCVA, best-corrected visual acuity; BRVO, branch retinal vein occlusion; IOP, intraocular pressure.

**Table 3:** Linear regression model of OCT measures and study variables.

Predictor	CMT	FM	WVA	SONT	IONT	TONT	NONT
	Standardized Estimate, *p*-Value						
**Age**	−0.03	0.12	−0.005	−0.479	−0.176	−0.24	−0.471
***p*-value**	0.823	0.627	0.984	**0.027**	0.459	0.3	**0.023**
**UCVA**	0.83	−0.022	0.082	−0.044	0.041	0.247	0.22
***p*-value**	0.806	0.89	0.621	0.742	0.782	0.094	0.09
**BCVA**	−1.26	0.123	0.074	0.32	0.341	−0.087	0.086
***p*-value**	0.836	0.489	0.684	0.054	0.068	0.623	0.582

Abbreviations: UCVA, uncorrected visual acuity; BCVA, best-corrected visual acuity; CMT; central macular thickness; FM, fovea minimum; WVA, whole-volume area; SONT, superior optic nerve thickness; IONT, inferior optic nerve thickness; TONT, temporal optic nerve thickness; NONT, nasal optic nerve thickness.

**Table 4:** OCT measures and study variables.

Outcomes OCT Measures	Study Group			Sex			Laterality			Type of Amblyopia		
	Amblyopia	Fellow	*p*-Value	Male	Female	*p*-Value	Left	Right	*p*-Value	Anisometropic	Strabismic	*p*-Value
**CMT**	267 (45.0)	266 (39.0)	0.832	278.1 (33.8)	251.9 (53.6)	**0.019**	270.0 (30.0)	262.3 (51.0)	0.8	273.6 (28.8)	222.5 (93.2)	**0.017**
**FM**	220 (41)	209 (41)	**0.047**	228.6 (33.6)	207.3 (46.3)	**0.007**	217.7 (33.7)	210.8 (47.1)	0.4	223.5 (28.5)	198.3 (84.5)	0.5
**WVA**	8.42 (0.83)	8.56 (0.61)	0.192	8.6 (0.6)	8.2 (1.0)	**0.01**	8.6 (0.5)	8.4 (0.9)	0.081	8.5 (0.5)	7.8 (1.8)	0.11
**SONT**	123 (26.0)	127 (20.0)	0.338	131.8 (17.5)	112.1 (30.1)	**0.004**	126.3 (22.6)	123.5 (23.7)	0.4	123.2 (22.7)	120.5 (44.5)	0.5
**IONT**	127 (26.0)	134 (20.0)	**0.026**	129.6 (24.0)	124.3 (28.4)	0.8	135.1 (22.2)	126.9 (24.1)	0.08	130.1 (20.3)	104.1 (43.5)	0.11
**TONT**	69 (11.0)	72 (13.0)	0.139	73.4 (12.4)	65.1 (6.9)	**0.017**	69.1 (10.5)	71.9 (13.2)	0.3	69.8 (9.8)	68.0 (17.3)	0.5
**NONT**	86 (18.0)	82 (14.0)	0.162	85.9 (16.3)	87.6 (19.3)	0.4	87.7 (15.4)	79.7 (16.2)	**0.002**	86.4 (14.1)	83.5 (30.8)	>0.9

Abbreviations: CMT, central macular thickness; FM, fovea minimum; WVA, whole-volume area; SONT, superior optic nerve thickness; IONT, inferior optic nerve thickness; TONT, temporal optic nerve thickness; NONT, nasal optic nerve thickness.

## Data Availability

The datasets used and analyzed during the current study are available from the corresponding author upon reasonable request owing to privacy and ethical restrictions.
